# Amphibious Miniature Soft Jumping Robot with On‐Demand In‐Flight Maneuver

**DOI:** 10.1002/advs.202207493

**Published:** 2023-04-25

**Authors:** Yibin Wang, Xingzhou Du, Huimin Zhang, Qian Zou, Junhui Law, Jiangfan Yu

**Affiliations:** ^1^ School of Science and Engineering The Chinese University of Hong Kong 518172 Shenzhen China; ^2^ Shenzhen Institute of Artificial Intelligence and Robotics for Society 518172 Shenzhen China; ^3^ Department of Mechanical and Industrial Engineering University of Toronto Toronto ON M5S 3G8 Canada; ^4^ School of Medicine The Chinese University of Hong Kong 518172 Shenzhen China

**Keywords:** jumping robot, magnetic actuation, small‐scale robot, soft materials, motion planning

## Abstract

In nature, some semiaquatic arthropods evolve biomechanics for jumping on the water surface with the controlled burst of kinetic energy. Emulating these creatures, miniature jumping robots deployable on the water surface have been developed, but few of them achieve the controllability comparable to biological systems. The limited controllability and agility of miniature robots constrain their applications, especially in the biomedical field where dexterous and precise manipulation is required. Herein, an insect‐scale magnetoelastic robot with improved controllability is designed. The robot can adaptively regulate its energy output to generate controllable jumping motion by tuning magnetic and elastic strain energy. Dynamic and kinematic models are developed to predict the jumping trajectories of the robot. On‐demand actuation can thus be applied to precisely control the pose and motion of the robot during the flight phase. The robot is also capable of making adaptive amphibious locomotion and performing various tasks with integrated functional modules.

## Graphical abstract

A part of semiaquatic insects can jump on the water surface leveraging surface tension. Inspired by these insects, this work presents a miniature magnetic soft robot with the capabilities of water‐surface jump, on‐demand in‐flight maneuver, and amphibious multimodal locomotion. Dynamic model and kinematic models are developed, and based on them, the motion of the robot can be precisely predicted and controlled.

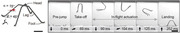

## 1 Introduction

Jumping ability emerged during biological evolution endows the creatures owning it with larger living space and faster movement for predation and escaping from predators.^[^ ^1^ ^]^ The optimized muscle dynamics after natural selection results in the efficient jumping behaviors of the creatures.^[^ ^2^, ^3^, ^4^ ^]^ Emulating the biomechanics of these jumping creatures, various engineered jumpers capable of taking off from the solid ground and the water surface have been developed.^[^ ^5^, ^6^, ^7^, ^8^, ^9^, ^10^, ^11^, ^12^, ^13^, ^14^, ^15^, ^16^, ^17^, ^18^, ^19^, ^20^, ^21^, ^22^ ^]^ The use of high‐power, high‐energy motors, and transmission systems provides terrestrial jumpers with large driving forces and considerable jumping performance. Meanwhile, different criteria of design are required for jumpers on the water surface, including the superhydrophobic surface, low body mass, and efficient stroke method mimicking water jumping insects.^[^ ^23^, ^24^, ^25^, ^26^, ^27^ ^]^ Spring latch systems and motor‐spring systems are widely used as power sources of the jumping robots on the water surface.^[^ ^8^, ^16^, ^17^, ^18^, ^19^ ^]^ Spring‐latch systems can be implemented on small‐scaled robots for its small mass and high power density, however, reloading is required after each spring release for jumping, which limits its continuous operation. Even though motor‐spring systems enable the continuous jump of robots, the comparatively low power density and high body mass decrease the jumping height. Magnetic actuation strategy is promising in addressing the limitations of the existing water‐surface jumping robots to achieve higher controllability and agility.

Magnetic robots can be actuated wirelessly and rapidly by the external magnetic field.^[^ ^28^, ^29^, ^30^, ^31^, ^32^, ^33^, ^34^ ^]^ Without implementing bulky on‐board power sources and mechanical transmission systems, both the mass and size of the magnetic soft robots can be minimized, and moreover, continuous actuation of the robots can be realized. One of the major applications of miniature magnetic robots is the biomedical application because they can non‐invasively access and navigate in difficult‐to‐reach areas inside the human body.^[^ ^35^, ^36^, ^37^, ^38^, ^39^, ^40^, ^41^ ^]^ To date, agile magnetic soft robots with versatile locomotion modes have been developed, such as walking, swimming, crawling and rolling robots.^[^ ^28^, ^42^, ^43^, ^44^, ^45^, ^46^, ^47^ ^]^ The jumping motions of the magnetic robots are also achieved, for instance, a magneto‐elastic millimeter‐scale robot is developed that can jump over an obstacle by imparting impulsive impact on a solid surface through rapid shape change.^[^ ^28^ ^]^ However, none of them can achieve both on‐ground and on‐water locomotion and jump. It remains challenging for the magnetic soft robot to adaptively navigate across the unstructured aquatic‐terrestrial environment, which is often seen in biomedical applications. Moreover, in real‐world applications, both the controllability and agility of the miniature magnetic robot need to be further improved for precise positioning and efficient functioning.

Herein, we propose a miniature soft robot that is capable of jumping on the water surface with on‐demand in‐flight maneuver. The surface of the robot is engineered to be superhydrophobic, and by performing a striking motion on the water surface, the robot exploits the water surface tension for controlled jumping, mimicking the water strider in nature. The dynamic model is established for the jumping process, and controlled momentum transfer on the water surface is also investigated. The kinematic model is developed for predicting and controlling jumping trajectories. Based on the models, the strategy for on‐demand in‐flight maneuver is established. Moreover, multimodal locomotion and potential applications of the robots are further explored and demonstrated.

## 2 Results

**Figure 1:**
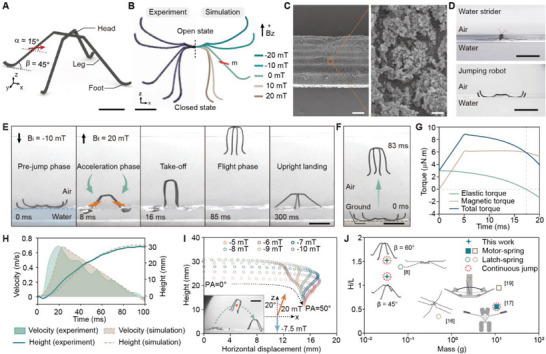
Controlled jump of the soft jumping robot. A) The soft jumping robot with a leg angle (*β*) of 45° and a magnetization angle (*α*) of 15°. The red arrow indicates the magnetization direction of the leg (scale bar, 5 mm). B) The comparison between the experimental deformations of the robot and the corresponding simulation results in static magnetic fields (***B***_**z**_) with different directions and magnitudes (scale bar, 5 mm). C) SEM images of the robot surface with SiO_2_ nanoparticles coating (scale bars, left: 100 µm, right: 500 nm). D) A real water strider floating on the water surface and a jumping robot sprawling on the water surface under a magnetic field of ‐10 mT (scale bars, 1 cm). E) The robot jumps from the water surface (scale bar, 1 cm). The red arrows indicate the magnetization direction, and the green arrows indicate the stroke direction of the legs. F) The robot jumps from the solid ground (scale bar, 1 cm). The strength of the applied initial magnetic field (***B***_**i**_) and the take‐off magnetic field (***B***_**t**_) are ‐10 mT and 20 mT, respectively. G) The simulation results of the torque profiles exerted on the robot legs in the acceleration phase. H) Time‐dependent velocity and height profiles of the robot jumping on the water surface. I) Peak point positions of the forward jumping trajectories when the robot is actuated by initial magnetic fields with different strengths and take‐off magnetic fields with different pitch angles (*σ*). Along each curve, the corresponding pitch angle for each data point increase from 0° to 50° with an increment of 1°. The robot performs a forward jump is shown in the inset (scale bar, 1 cm). The green dashed line indicates the trajectory, and the red dot indicates the peak point position of the trajectory. The blue arrow indicates the direction of the initial magnetic field, and the orange arrow indicates the direction of the take‐off magnetic field. J) The summary of the jumping performances of previously reported jumping robots on the water surface.

The robot is assembled from four magnetoelastic legs made of silicone rubber (PDMS) embedded with neodymium‐iron‐boron (NdFeB) microparticles (**Figure** **1**A). The legs are fabricated through molding, surface coating, aging, and magnetization (Figure S1, Supporting Information, Experimental Section). All the legs are magnetized to have the same magnetization angle (*α*), which is defined as the angle between the magnetization direction and the horizontal plane (Figure S1, Supporting Information). Actuated by external magnetic fields with different directions and magnitudes, the magnetoelastic robot (*α* = 15°) deforms and presents time‐varying configurations. Its experimental and simulated configurations are presented in Figure 1B (Experimental Section). In a magnetic field along the positive direction of the z‐axis, the legs rotate downward to a closed state, while rotating upward to an open state when reversing the field

direction. To fully exploit the water surface tension for jumping, superhydrophobic SiO_2_ nanoparticles are coated on the uncured PDMS surface, and after subsequent fully curing and aging, the SiO_2_ nanoparticles are fixed on the surface. The SEM image of the robot surface reveals the uniform coating of SiO_2_ nanoparticles forming a microporous superhydrophobic layer (Figure 1C). The superhydrophobic property of the coated surface is characterized (Note S1 and Figure S3, Supporting Information) and its influence on the jumping capability of the robot is discussed (Note S2, Supporting Information). With the superhydrophobic layer, the robot can thus sprawl on the water surface supported by surface tension mimicking a water strider (Figure 1D).

### 2.1 Controlled Jump of the Soft Jumping Robot

To characterize the jumping performance, the robot with a magnetization angle of 15° (*α* = 15°) and a leg angle of 45° (*β* = 45°) is investigated, where the leg angle is defined as the angle between the leg and the horizontal plane (Figure 1A). In the pre‐jump phase, the robot is first actuated to sprawl on the water surface to enlarge the stroke range of the legs for the subsequent jumping. In the meantime, the elastic strain energy accumulates at the deformed elastic hinge, which can be exploited to enhance the jumping performance of the robot (Figure S5, Supporting Information). Upon applying a take‐off magnetic field, the robot rapidly rotates its legs towards the water surface driven by the magnetic torque and the elastic torque. Due to the superhydrophobic nature of the legs, the water is pushed downward, forming dimples that provide vertical propulsion for the robot. After the acceleration period, the robot gains sufficient momentum to take off from the water surface and reaches a jumping height of 30 mm (Figure 1E and Movie S1, Supporting Information). With the same actuation parameters, the robot can also be powered to jump to 29 mm on a solid ground (Figure 1F and Movie S1, Supporting Information).

A dynamic model quantitatively analyzing the jumping dynamics and the corresponding jumping performance is developed (Note S3, Supporting Information), where the hybrid driving source for the system consisting of the magnetic torque and the elastic torque is considered. The model also describes the dynamic processes involved in the jumping process: the rotation of the robot leg, the growth of the dimple, the resultant propulsion force generation, and the consequent jumping motion. Based on the dynamic model, the time‐dependent magnetic torque, elastic torque, and total torque exerted on the legs of the robot in the jumping process presented in Figure 1E are simulated (Figure 1G). Instead of a sudden rise, the magnetic torque at first increases with time, because the applied magnetic field strength also gradually increases with the existence of the electromagnetic impedance. Afterward, the magnetic torque decreases, due to the reduced phase angle difference between the magnetization of the robot legs and the external magnetic field. Meanwhile, the elastic torque generated from strain energy peaks at the beginning, and decades as the shape deformation recovers. As the robot transits from the open state to the closed state, the elastic torque decreases, and when a negative value is reached, the direction of the elastic torque reverses. The elastic torque and magnetic torque compensate for each other, and an overall high level of energy input to the dynamic system is achieved, as shown by the profile of the total torque. The simulation results of the time‐dependent velocity profile and jumping height are obtained, which match well with the experimental results (Figure 1H). The results indicate that the jumping dynamics and the position of the robot during the flight phase can be precisely modeled and predicted using our proposed model.

To further evaluate how the elastic deformation contributes to the jumping dynamics, a robot with free linkage to eliminate the elastic strain energy and a robot with a leg angle of 60° to increase the stored elastic strain energy in the pre‐jump phase are prepared (Note S4, Supporting Information). When actuated by a take‐off magnetic field *B*_t_ with a field strength of 20 mT, the maximum jumping height of the robot with free linkage is 28 mm (Figure S9, Supporting Information), which is lower than that of the robot with an elastic hinge (30 mm), while the jumping height of the robot with a leg angle of 60° is increased to 31 mm (Figure S10, Supporting Information). The results indicate that the elastic strain energy stored in the deformed soft body facilitates the jumping dynamics. When the take‐off magnetic field strength is increased to 30 mT, the jumping height of the robot with a leg angle of 60° can be further increased to 38 mm.

Besides the vertical jump, the robot can also perform the forward jump (Figure 1I and Movie S1, Supporting Information). By adjusting the strength of the initial magnetic field and the pitch angle of the take‐off magnetic field, the control of the forward jumping trajectories is demonstrated (Figure S12, Supporting Information). A data model is then fitted to predict the forward jumping trajectories (Note S5, Supporting Information) and the positions of their peak points (Figure 1I). The accessible region of the robot via a forward jump can thus be mapped (Figure S15, Supporting Information). The jumping performances of the robot we design and other jumping robots on the water surface are evaluated by comparing the ratio of the jumping height H to the body length L (Figure 1J).^[^ ^8^, ^16^, ^17^, ^19^ ^]^ The robot we design has the smallest mass and the highest jumping performance based on this criterion. The key parameters, including jumping height, body length, and mass are summarized in Table S1 (Supporting Information).

### 2.2 Jumping Performance of the Soft Jumping Robot

**Figure 2:**
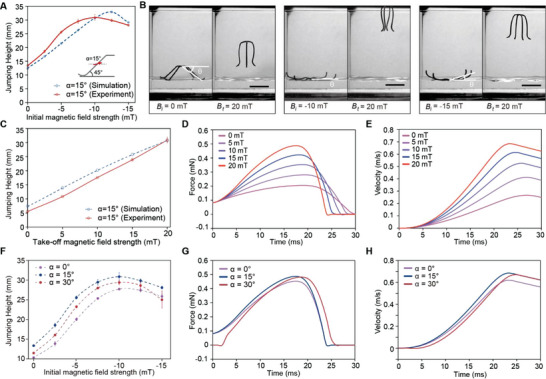
Jumping performance of the soft jumping robot. A) The jumping height of the soft jumping robot with a leg angle (*β*) of 45° and a magnetization angle (*α*) of 15° as a function of the initial magnetic field strength. B) The jumping process of the soft jumping robot with different initial magnetic fields (*B_i_* = {0 mT, ‐10 mT, ‐15 mT}). C) The jumping height of the robot (*α* = 15°, *β* = 45°) as a function of the take‐off magnetic field strength. D,E) The force profiles (d) and velocity profiles (e) obtained from numerical simulation corresponding to the jumping processes with different take‐off magnetic fields. F) The jumping height of the robots (*α* = {0°, 15°, 30°}, *β* = 45°) as functions of the initial magnetic field strengths. G) The force profiles and H) velocity profiles obtained from numerical simulation when the three robots are actuated by an initial magnetic field with a strength of ‐10 mT and a take‐off magnetic field with a strength of 20 mT.

The jumping dynamics is further investigated by adjusting the initial magnetic field strength, the take‐off magnetic field strength, and the magnetization angle of the robot. To investigate the influence of the initial magnetic field ***B***_**i**_ on the jumping performance, jumping trials are performed with different ***B***_**i**_ and a fixed ***B***_**t**_. The experimental results are plotted in **Figure** **2**A. The robot with a leg angle (*β*) of 45° and a magnetization angle (*α*) of 15° is applied in the tests. The jumping height of the robot increases as ***B***_**i**_ decreases from 0 to ‐10 mT. The reason is twofold. When ***B***_**i**_ decreases, the robot has a larger shape deformation, and the legs have a longer stroke range and acceleration process. Higher kinetic energy is thus imported into the system. Moreover, higher elastic strain energy is also stored in the pre‐jump phase due to the larger deformation of the robot (Figure 2B). The resulting higher energy input enhances the jumping performance. As ***B***_**i**_ further decreases to ‐12.5 mT and ‐15 mT, the jumping height decreases. In these cases, the robot legs raise above the water surface in the pre‐jump phase (Figure 2B). Therefore, the legs accelerate in the air, and then beat the water surface with a relatively large initial speed, which makes the water retreat rapidly and loses contact with the legs, undermining the energy efficiency. To quantitatively analyze the jumping process, the torque profiles, force profiles, and velocity profiles are obtained from the numerical simulation (Figure S16, Supporting Information). Based on the simulation results, the maximum energy input is achieved when the robot is actuated by an initial magnetic field with a strength of ‐12.5 mT. In the experiment, the maximum jumping height is achieved when *B_i_* = ‐10 mT, which indicates the maximum momentum transfer achieved. When the initial magnetic field strength decreases from ‐10 mT to ‐12.5 mT, the energy dissipation increases, resulting in the lower energy efficiency despite more energy is imported into the dynamic system.

The influence of the take‐off magnetic field ***B***_**t**_ on the jumping performance is evaluated by conducting jumping tests with the fixed initial magnetic field (*B_i_* = ‐10 mT) and different take‐off magnetic fields (*B_t_* = {5 mT, 10 mT, 15 mT, 20 mT}). The change in ***B***_**t**_ directly influences the magnetic torque according to ***τ***_***m***_ = ***m*** × ***B***. The experimental and simulation results of the jumping height with different take‐off magnetic fields ***B***_**t**_ is plotted in Figure 2C. As indicated by the results, in the specific range of magnetic field strength (0–20 mT), the jumping height increases almost in a linear manner with the take‐off magnetic field strength. The corresponding force profiles obtained from the simulation is shown in Figure 2D. As the strength of the take‐off magnetic field increases, the work done by the propulsion force increases as indicated by the coverage areas of the force profiles. Higher take‐off velocities can thus be obtained (Figure 2E).

To understand the influence of the magnetization angle of the robot on its jumping performance, robots with different magnetization angles (*α* = 0°, 15°, 30°) are prepared. The robots are fabricated following the process described in Figure S1 (Supporting Information). To characterize their jumping performance, each robot is actuated by a fixed take‐off magnetic field (*B_t_* = 20 mT) and initial magnetic fields with different strengths ranging from ‐15 to 0 mT. The results indicate that the three designs with different magnetization angles (*α* = 0°, 15°, 30°) have the maximum jumping height when *B_i_* = ‐10 mT. With the same initial magnetic field, the robot with a magnetization angle of 15° has better jumping performances compared with that of the other two designs with magnetization angles of 0° and 30° (Figure 2F).

The force and velocity profiles of the jumping processes with an initial magnetic field strength of ‐10 mT are simulated as shown in Figure 2G,H, respectively. The results indicate that the robot with a magnetization angle of 15° has the highest propulsion force throughout the acceleration phase compared with that of the other two designs. The highest take‐off velocity is also obtained by the robot with a magnetization angle of 15° (Figure 2H). Note that although the force profile of the robot with a 30° magnetization angle is comparable to that of the robot with a 15° magnetization angle, its take‐off velocity is lower. This can be explained by their different configurations in the pre‐jump phase. When *B_i_* = ‐10 mT, the robot (*α* = 30°) has a larger deformation with its legs rising above the water surface in the pre‐jump phase, resulting in higher energy dissipation in the subsequent acceleration phase.

### 2.3 In‐Flight Maneuver of the Jumping Robot

**Figure 3:**
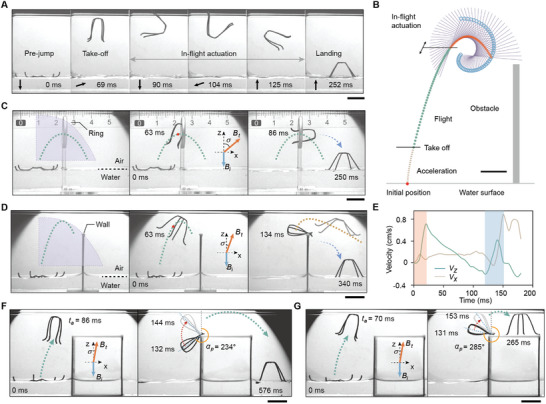
In‐flight maneuver of the jumping robot. A) In‐flight actuation enables a 360° backflip of the robot on the water surface (scale bar, 1 cm). The black arrows indicate the directions of the applied magnetic field. B) Trajectory and pose control of the robot for precise in‐flight actuation (scale bar, 5 mm). The lines caped with the blue circles indicate the long axis of the robot, and the blue circles indicate the position of the robot head. C) The robot jumps through a ring‐like obstacle with in‐flight pose control (scale bar, 1 cm). D) The robot jumps over a wall above the water surface through in‐flight energy restoration (scale bar, 1 cm). The brown dashed line presents the trajectory of the robot after reacceleration. E) The velocities of the robot in x‐direction and z‐direction when jumping over the wall above the water surface. F) The robot jumps over a stage when the in‐flight actuation starts at 86 ms (scale bar, 1 cm). The red arrow indicates the reacceleration range. G) The robot jumps onto a stage when the in‐flight actuation starts at 70 ms (scale bar, 1 cm). The blue and orange arrows indicate the direction of the initial magnetic field and the take‐off magnetic field, respectively. The purple region represents the accessible region of the robot, the green dashed line represents the planned trajectory of the robot. The red points indicate the positions of the robot when the in‐flight actuation starts.

The dynamics model and kinematics model we develop enable the precise in‐flight maneuver of the robot. By applying rotating magnetic fields with specific frequency and duration in the flight phase, the robot can perform 360° and 720° backflips after taking off from the water surface vertically (**Figure** **3**A, Figure S17 and Movie S2, Supporting Information). Combing the forward jump and the subsequent in‐flight backflip, the robot is able to perform controllable somersaults. Actuated by the programmed magnetic field sequence, the robot takes off from the water surface following the planned trajectory. Upon applying the in‐flight actuation, the robot starts rotating, while its mass center still follows the planned trajectory. A kinematic model is developed to predict the somersaulting trajectory and the pose of the robot in the flight phase (Figure 3B and Note S5, Supporting Information). Based on the desired pose and in‐flight position of the robot, the in‐flight actuation can be tailored according to the model. The robot can pass through a ring‐like obstacle using the proposed predictive model (Figure 3C and Movie S2, Supporting Information). A forward jumping trajectory passing through the center of the ring is first planned for the robot. Since the diameter of the ring is smaller than the maximum body height of the robot, the robot would be stuck when only the planned forward jump is performed (Figure S18, Supporting Information). With the programmed in‐flight actuation (Figure 3C, 63 ms), the robot adjusts its in‐flight pose and crosses the ring with the long axis of its body normal to the plane of the ring (Figure 3C, 86 ms).

The robot can also interact with the surrounding objects to perform in‐flight reacceleration, and thus expand its accessible region. To jump over a high obstacle that cannot be overcome by a forward jump, a trajectory is first planned for the robot to jump towards the obstacle (Figure 3D). Actuated by a subsequent rotating magnetic field with a pre‐characterized optimal frequency of 14 Hz, the starting time of in‐flight rotation is precisely predicted to make the robot rotate and reach the obstacle with its curved feet. The kinetic energy is transformed into the elastic strain energy stored in the deformed body (Figure 3D, 134 ms). The reacceleration of the robot is triggered by the rotating magnetic field and the accumulated energy, and the robot can thus jump over the obstacle with an updated trajectory (Movie S2, Supporting Information). The time‐dependent velocity of the robot is plotted in Figure 3E. The rapid increase in the robot velocity from 0 to 18 ms (orange region) corresponds to the acceleration in the take‐off phase. In the reacceleration phase (127 to 150 ms, blue region), the velocity in the y‐direction first decreases, indicating the damping of the kinetic energy by the obstacle, and it then increases, revealing the angular acceleration enabled by the releasing of the elastic strain energy. By tuning the starting time of the in‐flight maneuver, the reacceleration can be controlled for the robot to either jump over or land on a stage. When the in‐flight maneuver starts at 86 ms (Figure 3F), the robot starts rotation at the peak point of the planned trajectory, and comes into contact with the stage after rotating 180° (Figure S19, Supporting Information). The robot deforms to the closed state and begins to reaccelerate at an initial pose angle (*α*_p_) of 234° (Figure 3F, 132 ms). The robot then takes off and jumps over the stage (Figure 3F, 144–576 ms). In order to land on the stage, the starting time of the in‐flight actuation is shifted earlier (Figure 3G, *t_a_* = 70 ms), resulting in a larger initial pose angle and shorter acceleration range for the reacceleration (Figure 3G, 131 ms). The restored energy is thus reduced, and the stable landing on the stage is achieved (Movie S2, Supporting Information).

### 2.4 Amphibious Multimodal Locomotion of the Robot

**Figure 4:**
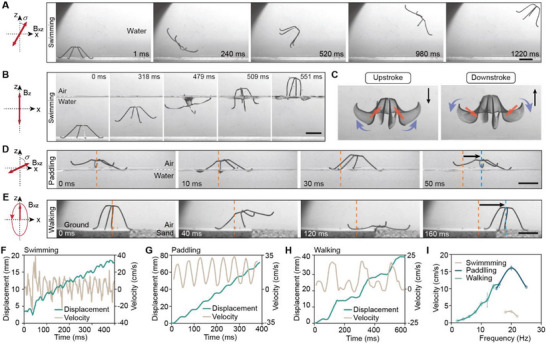
Amphibious multimodal locomotion of the robot. A) The robot swims in water with a desired direction. The motion is actuated by a 1D oscillating magnetic field with a strength of 20 mT, a frequency of 20 Hz, and a pitch angle of 20°. B) The robot swims in water and breaks the water‐air interface to float on the water surface. The motion is actuated by a 1D oscillating magnetic field with a strength of 20 mT and a frequency of 20 Hz. C) The upstroke and downstroke of the swimming gait. The black arrows indicate the directions of the magnetic fields, the red arrows indicate the magnetization directions, and the purple arrows represent the torques exerted on the robot legs. D) The robot paddles and moves on the water surface actuated by an oscillating magnetic field with a strength of 4 mT and a pitch angle of 60°. E) The robot walks from a flat surface to the sand actuated by an elliptically rotating magnetic field. F–H) The time‐dependent velocity and displacement of the robot when swimming in F) the water, G) paddling on the water surface, and H) walking on the ground, respectively. I) The velocity of the robot when performing different modes of locomotion. Scale bars: 1 cm.

The compliant nature of the robot enables different locomotion modes actuated by periodic time‐varying magnetic fields (Experimental Section). When immersed in water, the robot can swim along the desired direction by flapping its legs in a non‐reciprocal manner under a 1D oscillating magnetic field (**Figure** **4**A and Movie S3, Supporting Information). The robot can also break the water‐air interface (Figure 4B), attributing to its sufficient propulsion force and superhydrophobic surface. The swimming gait is composed of repeating upstrokes and downstrokes (Figure 4C). By applying a rotating magnetic field, the robot is deformed into the closed state and tumbles along a slope with a tilted angle of 30° (Figure S20 and Movie S3, Supporting Information). To accomplish efficient translational locomotion on the water surface, a paddling gait is developed for the robot (Figure 4D). Driven by a 1D oscillating magnetic field with a pitch angle of 60° and a frequency of 15 Hz, the robot can move on the water surface with a velocity of 16 cm s^−1^ (6 body length s^−1^). The paddling gait is composed of two steps, downstroke and upstroke. In downstroke, the legs paddle on the water surface to generate forward propulsion; while in upstroke, the legs lift above the water surface, avoiding the inversed motion and preparing for the next downstroke–upstroke cycle (Figure S22, Supporting Information). Mimicking inchworms, a walking gait is developed for the robot making translational locomotion on the solid ground (Figure 4E). In each walking cycle, the robot with a closed state first extends its front legs out to take a forward step, anchoring on its back feet. The robot then transforms from the open state to the closed state to pull its back legs forward (recovery step), anchoring on its front feet. The forward translational displacement is thus achieved (Figure S24, Supporting Information). The length of each step and the walking frequency can be tuned to achieve a precise and controllable motion on flat surfaces, sand and gradual slopes (Figure S25, Supporting Information). The displacement and velocity of the robot when performing the swimming, paddling and walking locomotion are analyzed. The oscillating velocity profile of the swimming mode with positive and negative values is resulted from the forward and backward displacements generated in the downstroke and the upstroke, respectively (Figure 4F). For the paddling locomotion, the robot speeds up when its legs strike the water surface for propulsion during the downstroke, while slowing down when gliding on the water surface in the upstroke. In this mode, the velocity of the robot maintains positive during the whole process (Figure 4G). In the walking mode, the velocity curve presents sequential peaks with small and large amplitudes (Figure 4H), which are resulted from the forward step (Figure 4E, 0–120 ms) and the recovery step (Figure 4E, 120–140 ms), respectively. Furthermore, the locomotion velocities of swimming, paddling, and walking actuated by magnetic fields with different frequencies are characterized (Figure 4I). The walking motion can be adopted in a wide range of frequencies (0 to 16 Hz). The threshold frequency for paddling and swimming are 15 and 18 Hz, respectively, below which the robot performs uncontrollable movements. Combining the developed gaits, we realize the on‐demand locomotion of the robot in an aquatic‐terrestrial environment, and the transitions among the motion gaits are applied adaptively (Figure S26, Supporting Information).

### 2.5 Integration of Functional Modules

**Figure 5:**
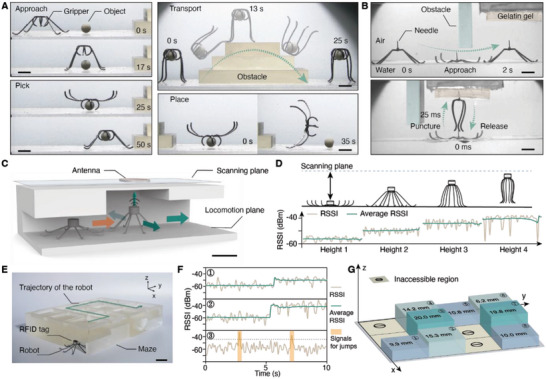
Integration of functional modules. A) A robot with a soft gripper picks, transports, and places a tiny object in water (scale bars, 5 mm). B) A robot with a needle overhead performs adaptive locomotion and targeted puncturing (scale bars, 5 mm). C) The schematics of the exploration strategy in an unknown maze‐like environment using the jumping robot (scale bar, 1 cm). The yellow arrow points from the previous to the current position of the robot. The green arrows show the exploration direction of the robot. D) The signal received by the antenna when the robot reaches different heights. The blue dashed line indicates the scanning plane where the antenna is located. E) The experimental image showing the robot ready to explore a maze (scale bar, 1 cm). The green dashed line indicates one of the possible pathways. F) The detected RSSI signal at positions 1, 2, and 3. The peaks labeled by the yellow region in position 3 indicate the signals received by the antenna when the robot jumps. The black dashed line indicates the average RSSI received by the antenna when the robot stands with the maximum height. G) The pathway and its height map inside the maze explored by the robot.

Furthermore, the robots with integrated functional modules are demonstrated. A soft gripper consisting of four curled parts with the same magnetization is integrated onto the robot, and it can perform precise pick‐and‐place tasks to non‐magnetic small objects (**Figure** **5**A and Movie S4, Supporting Information). Using a modified walking gait, the robot approaches the cargo (Experimental Section). The robot then lowers its body and the gripper is opened for the cargo. This motion is accomplished by designing the magnetization angle of the gripper the same as that of the robot legs. Therefore, when the robot stands up, its gripper will close simultaneously, and the cargo with a weight of 40 mg can be picked up. The robot carries the cargo and walks to a step‐like obstacle with a relatively shorter stride length, and then tumbles across it. The cargo is released after the robot arrives at the target position. Relying on the reconfiguration and jumping capability of the robot, we further demonstrate the targeted puncturing to a tissue‐mimicking gelatin gel using a robot with a needle assembled overhead. The robot is first navigated through a narrow gap above the water surface to the target region by performing a modified water surface locomotion (Experimental Section). With a subsequent vertical jump toward the gelatin gel, the needle on the robot pierces into the gel to the depth of 2.7 mm (Figure 5B and Movie S4, Supporting Information).

Integrated with a radiofrequency identification (RFID) tag, the robot can explore unknown environments through near‐field communication. An exploration strategy is developed for the robot to map the geometry of an unobservable maze‐like environment (Figure 5C). A peripheral antenna is located on the scanning plane above the robot to receive the signal, and the received signal strength indicator (RSSI) is used to estimate the distance between the RFID tag and the antenna (Figure 5D and Figure S27, Supporting Information). Since the robot can reach the roof of the maze by changing its body height or jumping from the ground, based on the change of RSSI, the height of the maze at different positions can be determined. Moreover, the RSSI decreases as the robot moves further from the antenna, the accessible direction of the robot inside the maze can thus be detected from a decaying signal. By repeating the detection procedure on the roof height and the accessible direction in each position of the maze, the 3D mapping of the region can be accomplished. In the experiment, a maze with a single pathway and a detection robot are prepared (Figure 5E). The pathway consists of square blocks with different heights. In this case, the robot is assembled with 8 legs to enhance its loading capacity for the RFID tag. Adopting the exploration strategy, the robot is successfully navigated out from the maze, and the RSSI detected at each position inside the maze is recorded (Figure S28, Supporting Information). The RSSI profiles detected at positions 1,2 and 3 are presented in Figure 5F. In positions 1 and 2, since the roof heights are lower than the maximum body height of the robot, the stand‐up motions of the robot can be used to measure the heights, which generate the step signals. In block 3 with a higher roof, the robot is actuated to keep jumping to reach the roof, and the appeared signal peaks are used to determine the height. Based on the RSSI, the 3D geometry of the pathway is mapped (Figure 5G).

The adaptability of the robot to navigate hybrid aquatic‐terrestrial environments has made it a promising platform for biomedical applications in the gastrointestinal system. To validate the potential biomedical application of the robot, we demonstrated the delivery of a mucoadhesive patch to a piece of porcine stomach tissue via water‐surface jumping (Figure S29, Supporting Information). We also anticipate that the robot could deliver patches to other hard‐to‐reach regions, such as the side wall of the stomach. Compared to the current medical procedure, the demonstrated method provides a non‐invasive approach to placing the drug‐loaded patch in the stomach.

## 3 Conclusion

In this work, we presented a soft‐bodied magnetoelastic robot capable of jumping on the water surface. The robot has enhanced jumping performances by leveraging the magnetic energy provided externally and the elastic strain energy pre‐stored in its deformed body. On‐demand in‐flight maneuver of the robot with time resolution in milliseconds is applied based on the proposed kinematic and dynamic models, and the pose and trajectory of the robot can be programmed to enable the controlled somersaults. Multimodal locomotion of the robot is developed for the adaptive movement in aquatic and terrestrial environments. The integration of functional modules enables the robot to perform tasks, including cargo delivery, targeted puncture, and environment exploration. The model‐driven actuation of the robot gives insights into the intelligent control of agile robots that move and interact with ambient environments. The design principle of leveraging elastic strain energy of the deformed elastomers has marked implications for soft power amplifying systems, and is transformative for the designing of onboard actuated soft robots. The design principle and control strategy proposed in this work will also provide inspiration for miniature amphibious robot and their potential applications in various fields.

## 4 Experimental Section

### Fabrication of the Soft Jumping Robots

The fabrication process includes molding, surface coating, aging, magnetization, and assembly (Figure S1A, Supporting Information). The material used for the soft robot was PDMS (SYLGARD 184 silicone elastomer kit, Dow Corning) embedded with neodymium‐iron‐boron (NdFeB) magnetic particles (MQFP‐15‐7, Magnequench). The monomer, crosslinker, and magnetic particles were blended according to a mass ratio of 5:1:6, and then mixed with a planetary mixer (MZ‐8, Thinky) at 2000 r.p.m. for 2 min. The mixture was then filled into a 3D‐printed PLA mold to fabricate the legs. After curing in a 55°C oven for 1.5 h, the partially cured legs were pulled out of the mold. SiO_2_ nanoparticles were then be pasted on the adhesive surface of the legs. After the surface coating, the legs were placed in a 125 °C oven for complete curing and aging. The fully cured legs were then magnetized to saturation with a magnetizer. By changing the magnetization direction, legs with magnetization angles (*α*) of 0°, 15°, and 30° were fabricated, where the magnetization angle is defined as the angle between the magnetization direction and the horizontal plane (Figure 1B). After magnetization, four legs with the same magnetization angle were glued together using uncured PDMS. The entire robot was then cured at 55 °C for 2 h to solidify, and an elastic hinge was formed at the robot head (Figure 1A). Following the same method, robots with magnetization angles of 0°, 15°, and 30° can be fabricated (Figure S1C, Supporting Information). The robot has a body mass of 33 mg and a density of 1.8 g cm^−3^. The detailed design parameters for the leg are shown in Figure S1D (Supporting Information).

### Finite Element Simulation

In the finite element simulation of the magnetoelastic actuators, the young's modulus *G* = 3.2 Mpa and magnetization *m* = 68.5 kA m^−1^ measured from the experiment are used as the input parameters (Note S1, Supporting Information). The strain of the elastic hinge of the robot in the open and closed states is visualized through the simulation (Figure S5, Supporting Information).

### Imaging Processing of the Robot

A high‐speed camera was used to capture the images of the jumping robots at 1000 frames per second. The captured images were processed in ImageJ to track the mass center of the robot and calculate the moving velocity.

### Actuation Strategy for the Multimodal Locomotion of the Robot

The different gaits for the multimodal locomotion of the robot are realized by the programmable time‐varying magnetic fields. For the swimming mode, a 1D oscillating magnetic field *B_z_* (*t*) = *A*sin (2*πft*) is applied, where*A* is the amplitude of the oscillating field, *f* is the frequency, and *t* is time. To actuate the upward swimming, the field with an amplitude larger than 20 mT and a frequency higher than 18 Hz is required to generate sufficient propulsion force against the drag force and the gravity. Applying the oscillating magnetic field with a pitch angle, the robot can swim along a desired direction (Figure 4A). The oscillating magnetic field can be expressed as:

(1) $$\begin{array}{c} B\left(t\right)=Asin\left(\gamma \right)sin\left(2\pi ft\right){\overset{⌢}{e}}_{x}+Acos\left(\gamma \right)sin\left(2\pi \mathrm{ft}\right){\overset{⌢}{e}}_{z} \end{array}$$

where *γ* is the pitch angle between the oscillating axis and the z‐axis, and $\begin{array}{c} {\overset{⌢}{e}}_{x} \end{array}$, $\begin{array}{c} {\overset{⌢}{e}}_{z} \end{array}$ stand for the unit vector along the x‐axis and z‐axis, respectively.

To realize the tumbling motion, a rotating magnetic field with a uniform strength is applied, which can be expressed as:

(2) $$\begin{array}{c} B\left(t\right)=Acos\left(2\pi ft\right){\overset{⌢}{e}}_{x}+Asin\left(2\pi ft\right){\overset{⌢}{e}}_{z} \end{array}$$

The 1D oscillating magnetic field in the same form as Equation (1) is applied for the paddling motion. With less drag force on the water–air interface compared with that inside the water, a magnetic field with a low amplitude (4 mT) is effective to actuate the robot to perform the paddling motion on the water surface. The influence of the applied field frequency and pitch angle on the paddling locomotion is investigated. Applying oscillating magnetic fields with a fixed amplitude (*B* = 4 mT), different frequencies ( *f*= {(15 Hz, 20 Hz, 25 Hz)}), and different pitch angles ( *γ*= {(15°, 30°, 45°, 60°, 75°)}), the velocity of paddling locomotion changes accordingly (Figure S23, Supporting Information). The result indicates that, with a pitch angle of 60°and a frequency of 20 Hz, the robot has the highest locomotion velocity. The robot steps out at a frequency of 25 Hz.

In the walking mode, an elliptically rotating magnetic field is applied. The field can be express as:

(3) $$\begin{array}{c} B\left(t\right)=\varepsilon Acos\left(2\pi ft\right){\overset{⌢}{e}}_{x} \end{array}+Asin\left(2\pi ft\right){\overset{⌢}{e}}_{z}$$

where $\begin{array}{c} \varepsilon \end{array}$ stands for the ratio between the minimum magnitude and maximum magnitude of the magnetic field. By adjusting the long‐axial direction of the elliptically rotating magnetic field perpendicular to a slope, the robot is able to move on the slope (Figure S25, Supporting Information). The resultant magnetic field for the actuation of the robot onto a slope can be expressed as:

(4) $$\begin{array}{c} B\left(t\right)=R\times \left(A\sin\left(2\pi ft\right){\overset{⌢}{e}}_{x}+A\sin\left(2\pi ft\right){\overset{⌢}{e}}_{z}\right) \end{array}$$

(5) $$R=\left[\begin{array}{cc} cos\delta & sin\delta \\ -sin\delta & cos\delta \end{array}\right]$$

where *δ* is the slope angle, and *R* is the transformation matrix.

### Magnetic Actuation for the Robot Integrated with Functional Modules

In order to realize the pick‐and‐place task, we proposed a modified walking gait. An offset is applied to the magnetic field for walking locomotion. The resultant magnetic field can be expressed as:

(6) $$\begin{array}{c} B\left(t\right)=\varepsilon Acos\left(2\pi ft\right){\overset{⌢}{e}}_{x} \end{array}+\left(Asin\left(2\pi ft\right)+s\right){\overset{⌢}{e}}_{z}$$

where *s* is the offset magnitude, and $\begin{array}{c} {\overset{⌢}{e}}_{x} \end{array}$, $\begin{array}{c} {\overset{⌢}{e}}_{z} \end{array}$ stand for the unit vector along the x‐axis and z‐axis, respectively. By tuning the offset magnitude*s*, the body height of the robot can be controlled to a fixed range during walking. With the modified walking gait, the robot approaches the cargo while avoiding the gripper from pushing the cargo away. The same modified gait is also used in the translational transportation of the cargo to guarantee the tight hold of cargo.

For the demonstration of puncture, a water surface locomotion mode for the robot passing through the narrow gap above the water surface with low body height is developed. The magnetic field consists of a rotating magnetic field to steer the legs for generating propulsion, and a static magnetic field *B_z_* = ‐10 mT to lower the body of the robot. The final magnetic field can be expressed in the same form as Equation (6). In Figure 5B, *s* is set as ‐10 mT, *A* is set as 5 mT, and *f* is set as 20 Hz.

### Statistical Analysis

The jumping height and velocity of the robot were measured from videos processed by ImageJ. The data are expressed as average values with standard errors. All the tests were performed in triplicate (*n* = 3), if not indicated differently.

## Conflict of Interest

The authors declare no conflict of interest.

## Author Contributions

Conceptualization: J.Y. and Y.W.; Methodology: J.Y., Y.W., X.D., H.Z., and Q.Z;. Investigation: Y.W., X.D., H.Z., and Q.Z.; Visualization: Y.W. and X.D.; Funding acquisition: J.Y.; Project administration: J.Y.; Supervision: J.Y.; Writing—original draft: Y.W., X.D., H.Z., and Q.Z.; Writing—review & editing: J.Y., Y.W., X.D., H.Z., Q.Z., and J.L.

## Supporting information

Supporting Information

Supplemental Movie 1

Supplemental Movie 2

Supplemental Movie 3

Supplemental Movie 4

## Data Availability

The data that support the findings of this study are available in the supplementary material of this article.
