# Supplementary material for: Amphibious Miniature Soft Jumping Robot with On‐Demand In‐Flight Maneuver
Source: Adv Sci (Weinh). 2023 Apr 25;10(18):2207493. doi: 10.1002/advs.202207493 (PMC10288233; doi:10.1002/advs.202207493)
Supplement: Supplementary file 1 — Supporting Information [file ADVS-10-2207493-s004.pdf]

ADVANCED SCIENCE
Open Access

# Supporting Information

for *Adv. Sci.*, DOI 10.1002/advs.202207493

Amphibious Miniature Soft Jumping Robot with On-Demand In-Flight Maneuver

*Yibin Wang, Xingzhou Du, Huimin Zhang, Qian Zou, Junhui Law and Jiangfan Yu**

# Supporting Information

**Amphibious miniature soft jumping robot with on-demand in-flight maneuver**

*Yibin Wang, Xingzhou Du, Huimin Zhang, Qian Zou, Junhui Law, Jiangfan Yu**

## Supplementary Text

Note 1 The characterization of the magnetoelastic material

To evaluate the mechanical property of the robot, we prepare a NdFeB/PDMS sheet using the same fabrication method of the robot for tensile tests. The Young's modulus of the material is measured as 3.2 MPa (Figure S2A). To measure the magnetization of the material, a cubic sample is prepared. The magnetic moment density (magnetization) of the sample is measured with a vibrating-sample magnetometer (7404, LakeShore) as 37.56 emu/g (Figure S2B). The SEM image of the cross-section of the robot leg shows the uniform distribution of NdFeB particles in the PDMS substrate (Figure S2C). The SEM image of the robot surface verifies the successful coating of $SiO_2$ nanoparticles (Figure 1C). To quantitatively analyze the hydrophobicity of the robot, we take contact angle tests on the film samples of pristine PDMS and $SiO_2$ coated PDMS, respectively. The pristine PDMS is hydrophobic with a contact angle of 105°, and $SiO_2$ coated PDMS is superhydrophobic with a contact angle of 160° (Figure S3).

Note 2 The influence of superhydrophobic surface on the jumping performance

To evaluate the influence of the superhydrophobic surface on the jumping performance of the robot, a robot without $SiO_2$ nanoparticle coating is prepared. Since PDMS is originally hydrophobic, the robot still manages to float on the water surface. However, the jumping performance of the robot is limited. When the robot is actuated by an initial magnetic field with a strength of -5 mT and a subsequent take-off magnetic field with a strength of 20 mT, it jumps to 14.7 mm (Figure S4A). It is noted that, the superhydrophobic robot fabricated by our proposed method can jump to a height of 25 mm with the same actuation parameters. Furthermore, when actuated by an initial magnetic field with a strength of -10 mT, the robot sprawls its legs on the water surface. However, in this case, a water film is formed connecting the robot legs, and the water surface tension traps the robot from taking off the water surface (Figure S4B).

Note 3 The dynamic model and numerical simulation of water-surface jump

A dynamic model is developed to better understand the jumping process. As illustrated in Figure S6, the robot is modeled as a pair of rigid rods connected by a free linkage. The mass and momentum inertia of each leg are $m$ and $I$, respectively. The angular position of the leg is represented by its position angle $\theta$, which is defined as the angle between the leg and the horizontal plane. The time-dependent magnetization direction can be represented as $\theta_m$. The leg can rotate around the linkage with an angular acceleration $\alpha$ and an angular velocity $\omega$ driven by the magnetic torque $\tau_m$ and elastic torque $\tau_e$. The magnetic torque $\tau_m$ can be calculated from the following equation:

$$\tau_m = |\boldsymbol{m}||\boldsymbol{B}| sin\left(\frac{\pi}{2} - \theta_m\right) \qquad (1)$$

where $\boldsymbol{m}$ is the magnetization and $\boldsymbol{B}$ is the external magnetic field. By taking the deformation test, the position angles of the leg that balance the elastic torque $\tau_e$ and magnetic torque $\tau_m$ in different static magnetic fields can be measured. The elastic torque $\tau_e$ as a

function of position angle $\theta$ can thus be fitted as $\tau_e = k_e\theta$ (Figure S7). The total torque can then be expressed as $\tau = \tau_m + \tau_e$. The water surface tension is molded as a spring-damper system with a spring constant $k_w$ of 0.375 N/m and a damping coefficient $c_w$ of 0.01 (*8*), and the dimple growth is modeled as the compression of the spring. Since the water surface tension force is the dominant factor in the water surface jumping, the propulsion force $F$ can thus be expressed as:

$$\mathrm{F} = -k_w d - c_w v_e \tag{2}$$

where $d$ is the dimple depth and $v_e$ is the growth rate of the dimple. As both $d$ and $v_e$ are dependent on the position of the leg, the propulsion force *F* can eventually be expressed as a function of $\theta$ and $h$, as $F = f(\theta, h)$. The propulsion force results in the upward motion of the robot with an acceleration $a$ and a velocity $v$ on its center of mass. Based on the dynamic model, the jumping process is then simulated with the following Velocity-Verlet method:

**Algorithm:** Numerical simulation of water-surface jumping

**Input**:
initial position angle of the leg $\theta$, initial height of the robot $h$, initial angular velocity of the leg $\omega$, and initial velocity of the robot $v$.
**Output**:
time-dependent position angle of the leg $\theta$, time-dependent height of the robot $h$

**for** $t = 0$ to T **do**
  **if** ($t$ = 0) **do**
    $\tau = \tau_m(\theta) + \tau_e(\theta)$
    $\alpha = \tau / I$
    $F = f(\theta, h)$
    $a = F / m$
  **else do**
    $\theta = \theta + \omega \times \Delta t + \frac{1}{2} \times \alpha \times \Delta t^2$
    $h = h + v \times \Delta t + \frac{1}{2} \times a \times \Delta t^2$
    $\tau = \tau_m(\theta) + \tau_e(\theta)$
    $\alpha' = \tau / I$
    $F = f(\theta, h)$
    $a' = F / m$
    $\omega = \omega + \frac{1}{2}(\alpha + \alpha') \times \Delta t$
    $v = v + \frac{1}{2}(a + a') \times \Delta t$
    $\alpha = \alpha'$
    $a = a'$
    $t = t + \Delta t$
    **return** $\theta$ and $h$
  **end if**
**end for**

Based on the numerical simulation, we can obtain the position, velocity, and acceleration of the robot at each time step (Figure S8).

Note 4 The jumping performance of the robot enhanced by pre-stored elastic strain energy

The robot with free linkage is fabricated following the process shown in Figure S9A. The legs are linked with a thin adhesive layer on the top. The layer is bendable but not elastic, and thus, it can be considered as a free linkage. Using this design, the robot is not able to stand on the water surface, it instead sprawls on the water surface (Figure S9B), and thus no initial magnetic field is required. The robot can be actuated directly by applying a take-off magnetic field. As shown in Figure S9C, with a take-off magnetic field of 20 mT, the robot is able to jump on the water surface to a height of 28 mm, which is lower than that of the robot with an elastic hinge (30 mm). The force profiles (Figure S9D) and velocity profiles (Figure S9E) of the robot with free hinge and the robot with elastic joint are simulated. Without the contribution from elastic strain energy at the beginning of acceleration, the propulsion force evolution of the robot with free linkage is slower than that of the robot with elastic joint, which results in a lower take-off velocity and lower jumping height (Figure S9F).

Informed by the torque profile shown in Figure 1H, it can be observed that the elastic torque undermines the dynamic process in the later stage of the acceleration when the robot transit from the open state to the closed state. We expect that when a robot with a larger leg angle jumps on the water surface, the transition from the open state to the closed state is delayed, resulting in less negative work done by the reversed elastic torque. A robot with a leg angle of 60° ($\beta = 60°$) is thus designed and fabricated to verify the assumption, where $\beta$ is defined as the angle between the leg and the horizontal plane (Figure S10A). With a larger leg angle, it requires a larger initial magnetic field to actuate the robot to sprawl on the water surface, indicating that higher elastic strain energy is stored in the pre-jump phase. When actuated by a take-off magnetic field with a strength of 20 mT, the robot can jump to a height of 31 mm on the water surface (Figure S10B), which is higher than that of the robot with a leg angle of 45º (30 mm). The improvement in jumping performance is mainly come from the increased elastic strain energy stored in the pre-jump phase, because the magnetic interaction involved in the jumping process keeps unchanged. When the take-off magnetic field strength is further increased to 30 mT, the jumping height of the robot with a leg angle of 60º is further increased to 38 mm, while the jumping height of the robot with a leg angle of 45º decreases to 29 mm (Figure S10C,D). For the jumping process of the robot with a leg angle of 60°, driven by the elastic strain energy at the beginning of the acceleration, the robot rapidly closes its legs inward and shallow dimples are generated, indicating an acceleration process with minimal energy dissipation (Figure S10E). For the robot with a leg angle of 45°, when jumping on water with a take-off magnetic field of 30 mT, the large torque induces the fast downward rotation of the legs, resulting in deep dimples that significantly decreases the energy efficiency (Figure S10F). The jumping performance thus decreases despite more energy being imported into the dynamic system. Although the jumping performance is improved, the robot with a leg angle of 60° is not stable in other locomotion modes, such as paddling and walking, because of its higher center of mass. Therefore, the robot with a leg angle of 45º and a magnetization angle of 15º is used for the following demonstrations in this work.

Note 5 The kinematic model for the trajectory and the in-flight pose prediction

The kinematic model for the forward jump is developed based on the experimental results. The robot with a magnetization angle of 15º and a leg angle of 45° is used for the forward jumping tests. Initial magnetic fields with different strengths ($B_i$ = {0 mT, -2.5 mT, -5 mT, -7.5 mT, -10 mT}), and take-off magnetic fields with different pitch angles ( $\sigma$ = {10°, 20°,

30°, 40°, 50°}) and fixed strength ($B_t$ = 20 mT) are applied. Pitch angle ($\sigma$) is defined as the angle between the take-off magnetic field direction and the positive direction of z-axis. In the kinematic model, we separate the forward jump into two phases: the acceleration phase and the flight phase (Figure S11). The acceleration phase is defined as the jumping process from the pre-jump phase to the time moment when the robot just leaves the water surface (take-off moment). The center of mass of the robot at the take-off moment is estimated, as:

$$x_0 = \frac{1}{2} \times l \times cos\left(\frac{\pi}{2} - \frac{1}{2}PA\right) \quad (3)$$

$$y_0 = \frac{1}{2} \times l \times sin\left(\frac{\pi}{2} - \frac{1}{2}PA\right) \quad (4)$$

where $x_0$, $y_0$ stand for the horizontal displacement and the height of the robot mass center, respectively, and $l$ represents the body height of the robot. The flight phase can then be considered as a free-falling starting from the take-off position with an initial take-off velocity. From the peak point position ($x_p$, $y_p$) of each trajectory, the take-off velocity can be calculated with the following equations:

$$v_y = \sqrt{2 \times g \times \left(y_p - y_0\right)} \quad (5)$$

$$t = \frac{v_y}{g} \quad (6)$$

$$v_x = \frac{x_p - x_0}{t} \quad (7)$$

The take-off velocities of the robot in forward jumps with different $B_i$ and $\sigma$ are calculated, as shown in Figure S12. On the basis of the results, the function of $v_x = f\left(B_i, \sigma\right)$ and $v_y = f\left(B_i, \sigma\right)$ can be fitted, as plotted in Figure S13. Therefore, with any actuation parameters $B_i$ and $\sigma$, we can predict the maximum jumping height $y_{max}$ and the corresponding horizontal displacement x based on the following equations:

$$t = v_y / g \quad (8)$$

$$y_{max} = y_0 + v_y^{\ 2} / 2g \quad (9)$$

$$x = x_0 + v_x \times t \quad (10)$$

The predicted maximum jumping height $y_{max}$ and the corresponding horizontal displacement x are shown in Figure S14. The forward jumping trajectories can thus be predicted, and by combing all the trajectories, we can map the accessible region of the robot via a forward jump in the 3D space (Figure S15).

Combining the forward jump and the in-flight actuation, the robot can perform somersault. The position of the robot during somersault can be predicted with the kinematic model of forward jump. The time-dependent pose angle ($\alpha_p$) of the robot during somersault, representing the angle between its body axis and the vertical direction, can be modeled with the following equation:

$$\alpha_p = \sigma + \omega_r \times \left(t - t_a\right) \quad (11)$$

where $\sigma$ is the pitch angle of the take-off magnetic field, $\omega_r$ is the average angular velocity of the rotation, and $t_a$ is the starting time of the in-flight actuation. With the kinematic model,

the trajectory of the somersault and the pose of the robot along the somersaulting trajectory can be predicted through simulation (Figure 3B).

**Supplementary Figures**

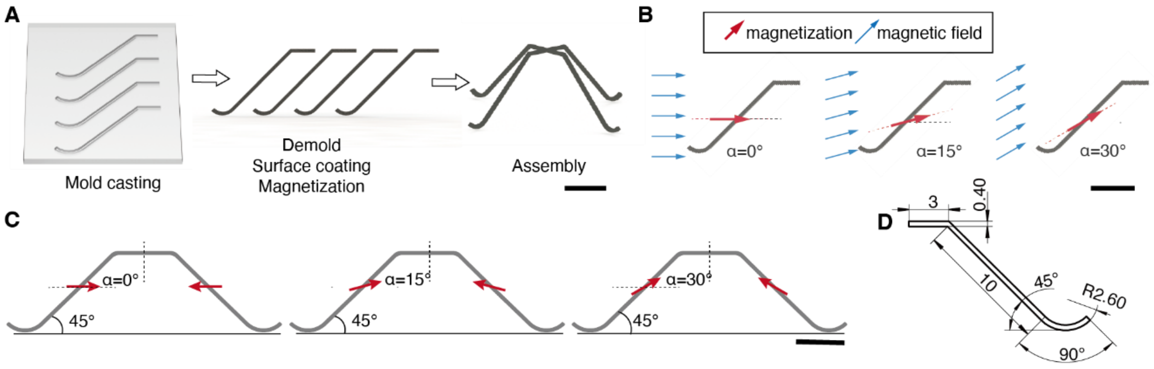


**Figure S1.**

**The fabrication process of the jumping robot.** A) The schematics of the fabrication process. B) The schematics of the magnetization process. The magnetization angle (α) is defined as the angle between the magnetization direction and the horizontal plane. C) The magnetization profile of the robot with different magnetization angle (α = 0°, 15°, 30°). d, The schematics of the robot leg. Scale bars, 5 mm.

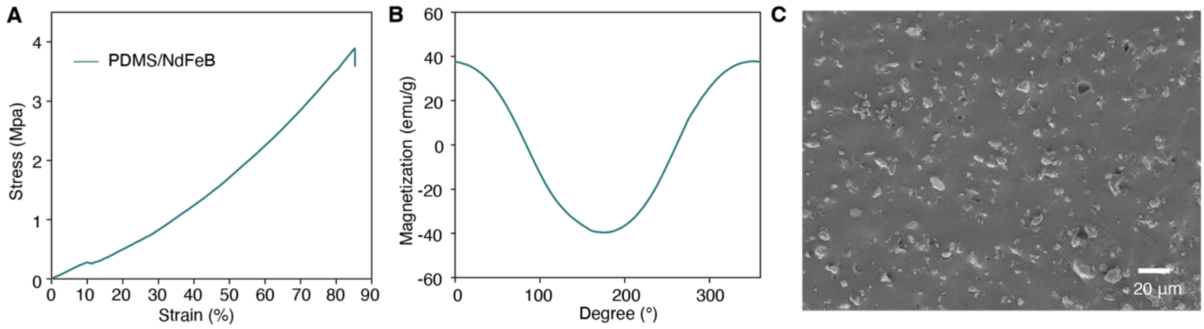


**Figure S2.**

**Characterization of the NdFeB/PDMS material.** A) Stress-strain curve of the NdFeB/PDMS sample. B) Magnetization values measured at different angular positions of the magnetized NdFeB/PDMS sample by the vibrating sample magnetometer (VSM). C) The SEM image of the cross-section of a robot leg. The NdFeB microparticles with average size of 5 μm is uniformly distributed in the PDMS matrix.

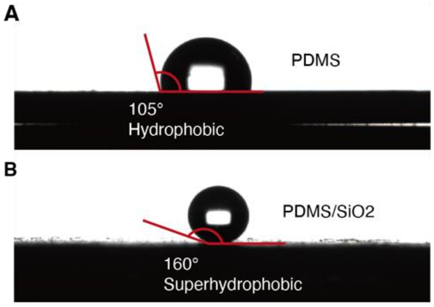


**Figure S3.**

**Characterization of the superhydrophobic surface of the robot**. A) Contact angle of water droplet on pristine PDMS film. B) Contact angle of water droplet on PDMS film with $SiO_2$ nanoparticle coating.

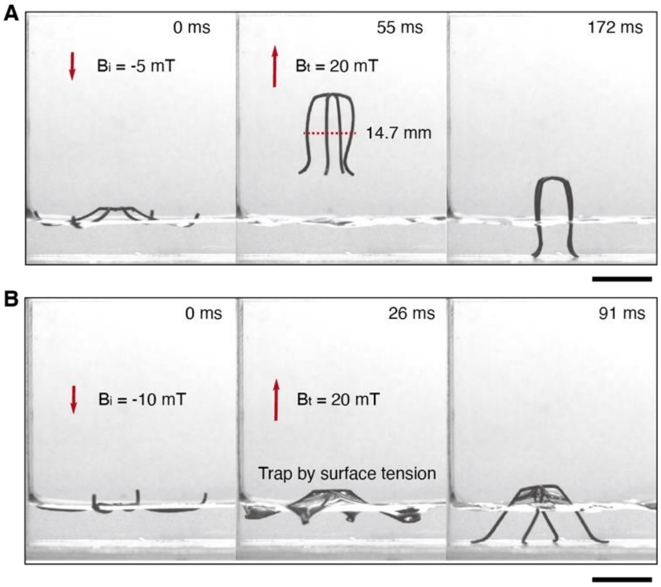


**Figure S4.**

**The jumping performance of the robot without a superhydrophobic surface.** A) The robot jumps on the water surface actuated by an initial magnetic field with a strength of -5 mT and a take-off magnetic field with a strength of 20 mT. B) The robot is trapped by surface tension under an initial magnetic field with strength of -10 mT. Scale bars, 1 cm

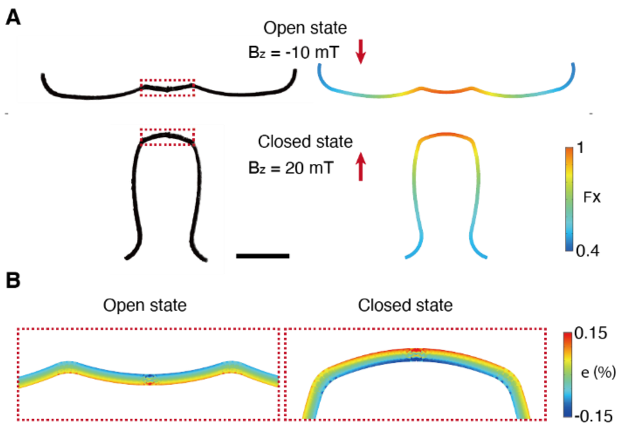


**Figure S5.**

**Finite element simulation.** A) Deformation of the robot under a static magnetic field $\boldsymbol{B_z}$ demonstrated by the experiments and the finite element simulations (scale bar, 1 cm). The elastic joint of the robot is labeled by the red dashed square. The color map indicates the deformation gradient. B) The strain of the elastic joint in the open state and the closed state.

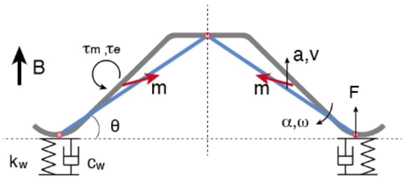


**Figure S6.**

**The dynamic model of the robot jumping on the water surface.** The robot is modeled by two rigid rods (indicated by two blue lines) rotating around a linkage. The red arrows indicate the magnetization direction of the modeled legs. The water surface is modeled by a spring-damper system with a spring constant of $k_w$ and a damping coefficient of $c_w$.

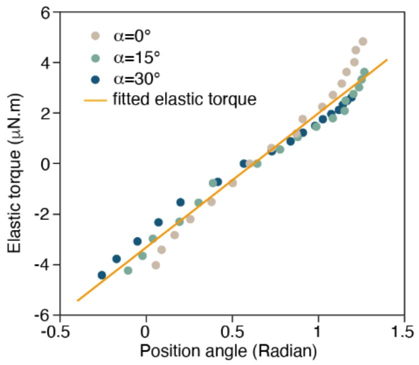


**Figure S7.**

**The fitted elastic torque as a function of the position angle.** The position angle $\theta$ is defined as the angle between the modeled robot leg and the horizontal plane.

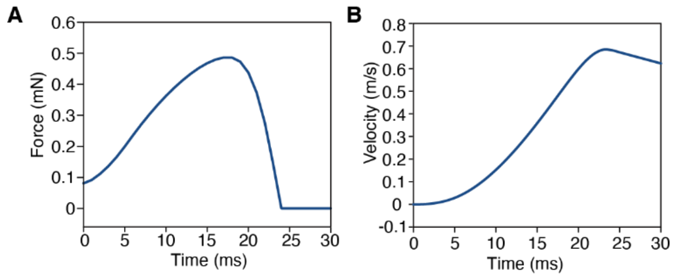


**Figure S8.**

**Numerical simulation of the water-surface jumping process.** A,B) The simulated time-dependent force profile (A) and the velocity profile (B) for the robot ($\alpha = 15°$, $\beta = 45°$) jumping with an initial magnetic field strength of -10 mT and a take-off magnetic field strength of 20 mT.

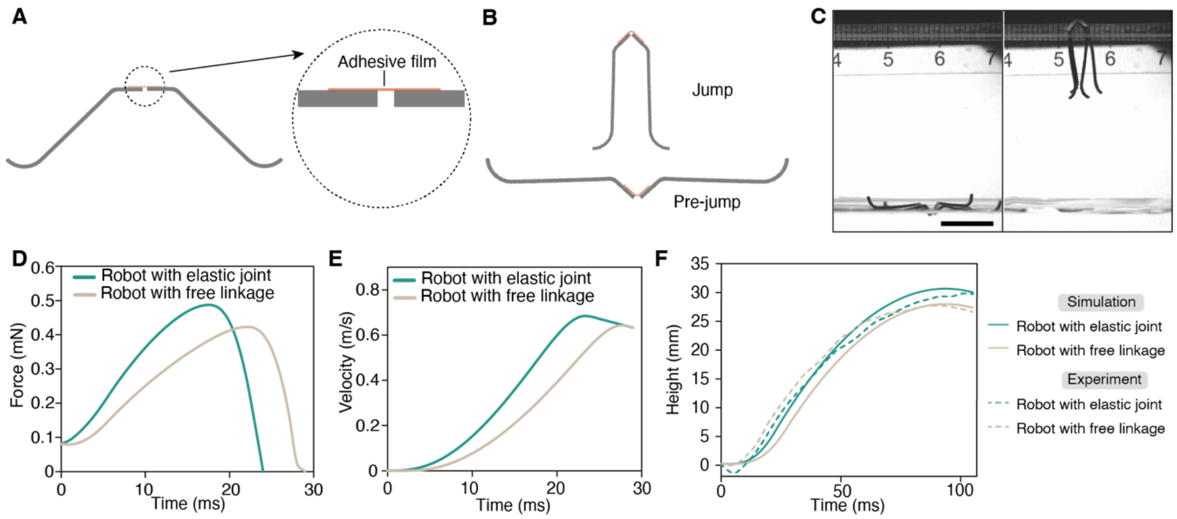


**Figure S9.**

**The jumping performance of the robot with a free linkage.** A) The schematics of the robot with free linkage. The zoom in region shows the connection part between the legs. B) The schematics of the jumping process for the robot with a free linkage. C) The robot with free linkage jumps on the water surface actuated by the take-off magnetic field with a strength of 20 mT (scale bar, 1 cm). D-E) Force profiles (D) and velocity profiles (E) obtained from the numerical simulation when the robots are actuated by the take-off magnetic field with a strength of 20 mT. F) The jumping height of the robot with a free linkage and the robot with an elastic joint.

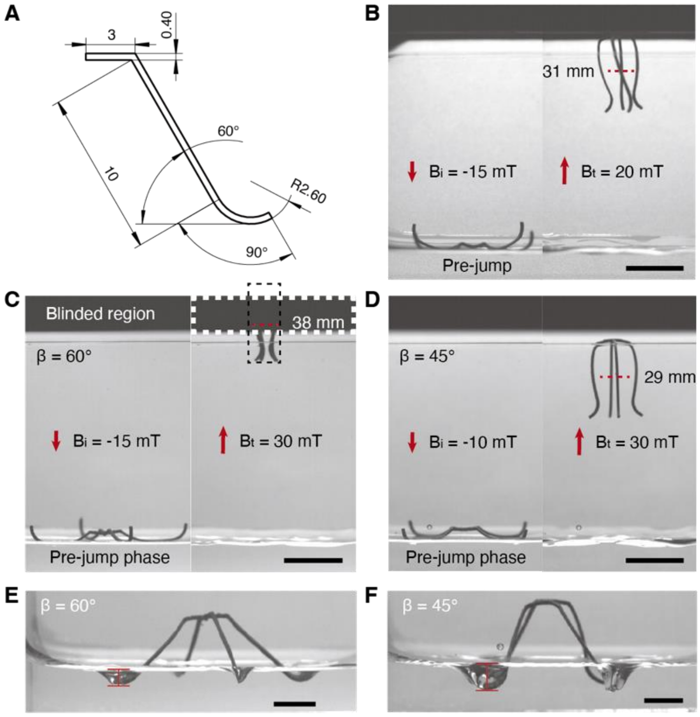


**Figure S10.**
**The jumping performance of the robot with a leg angle of 60°.** A) The schematic model of the robot with a leg angle ($\beta$) of 60°. B) The robot ($\beta$ = 60°) jumps on the water surface with $B_i = -15$ mT and $B_t = 20$ mT. The red dashed line indicates the height of the robot center. C) The robot ($\beta$ = 60°) jumps on the water surface with $B_i = -15$ mT and $B_t = 30$ mT. The white dashed square indicates the blinded region, and the black dashed square indicates the outline of the robot at the maximum jumping height. D) The robot ($\beta$ = 45°) jumps on the water surface with $B_i = -10$ mT and $B_t = 30$ mT. E-F) The maximum dimple depth generated by the robot with a leg angle of 60° (E) and the robot with a leg angle of 45° (F) when they are actuated by the take-off magnetic field with a strength of 30 mT. Scale bars: 1 cm.

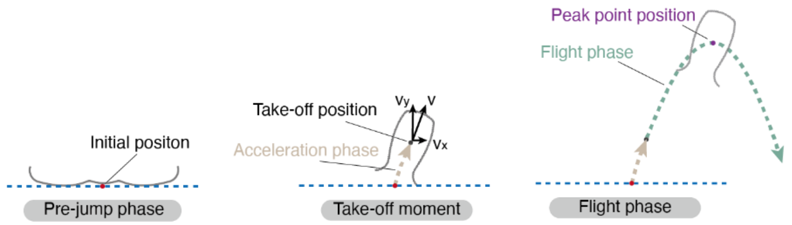


**Figure S11.**

**The schematics of the kinematic model for a forward jump.** The forward jump is separated into two stages, the acceleration phase and the flight phase.

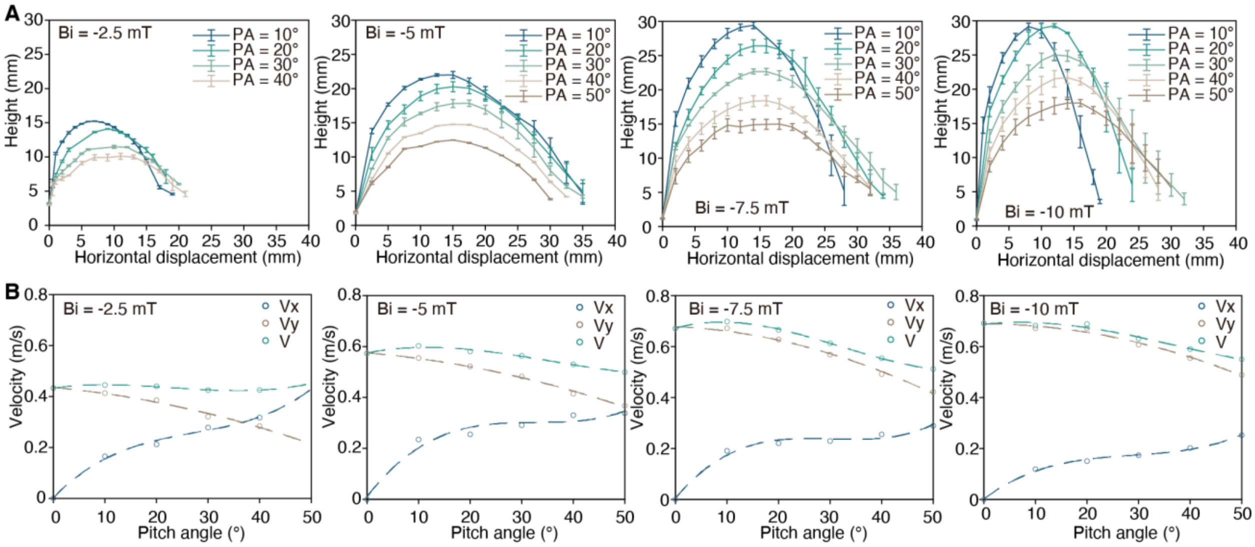


**Figure S12.**

**The trajectories and take-off velocities of forward jumps.** A) The forward jumping trajectories of the robot when it is actuated by initial magnetic fields with different strengths and take-off magnetic fields with different pitch angles. B) The take-off velocity of the robot when it is actuated by initial magnetic fields with different strengths and take-off magnetic fields with different pitch angles. The x-component and y-component of the take-off velocity $\boldsymbol{v}$ are represented with $v_x$ and $v_y$, respectively.

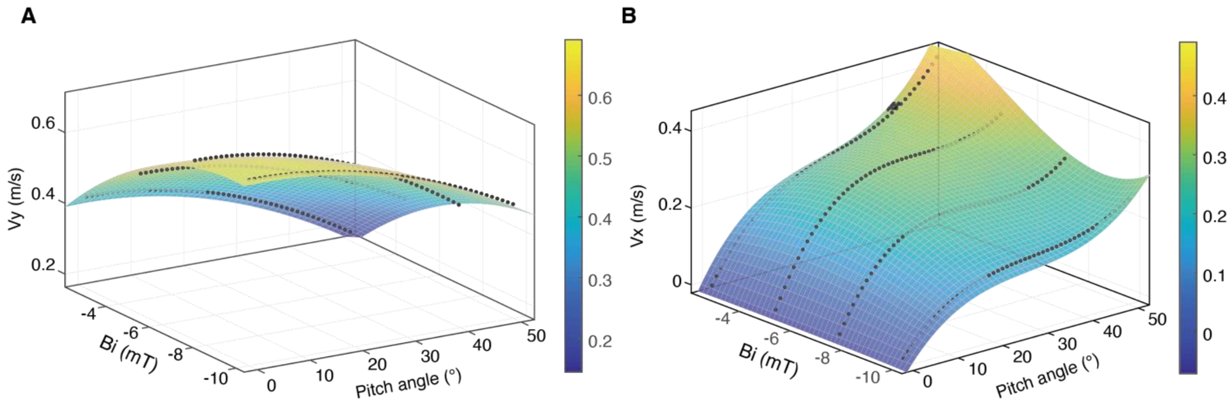


**Figure S13.**

**The fitted data model of the take-off velocity of the robot.** A,B) The fitted y component of the take-off velocity ($v_y$) (A) and the fitted x component of the take-off velocity ($v_x$) (B) as functions of the initial magnetic field strength ($B_i$) and the pitch angle (PA) of the take-off magnetic field.

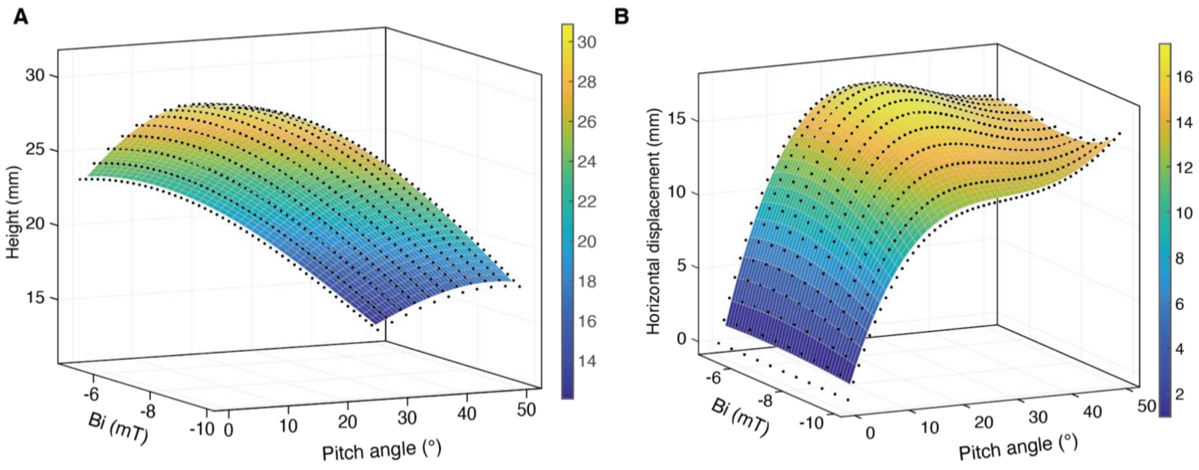


**Figure S14.**

**The fitted data model of the peak point positions of different forward jumping trajectories.** A,B) The predicted maximum heights of the forward jumping trajectories (A) and the corresponding horizontal displacements in the x direction (B) when different magnetic fields are applied for the forward jumps. The initial magnetic field strength ranges from -10 mT to -5 mT with an increment of 0.5 mT, and the pitch angle of the take-off magnetic field ranges from 0° to 50° with an increment of 1°.

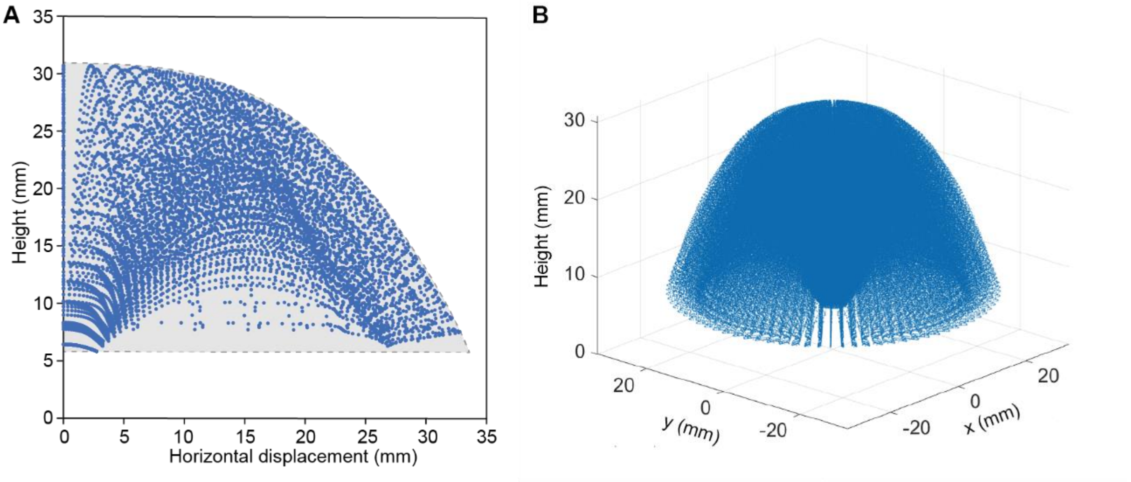


**Figure S15.**

**The accessible region of the robot.** A) The accessible region of the robot in the x-z plane. The region is mapped by stacking the possible forward jumping trajectories. The blue points represent the simulated trajectories and the gray region represents the maximum accessible region. B) The accessible 3-dimensional space of the robot via forward jumps.

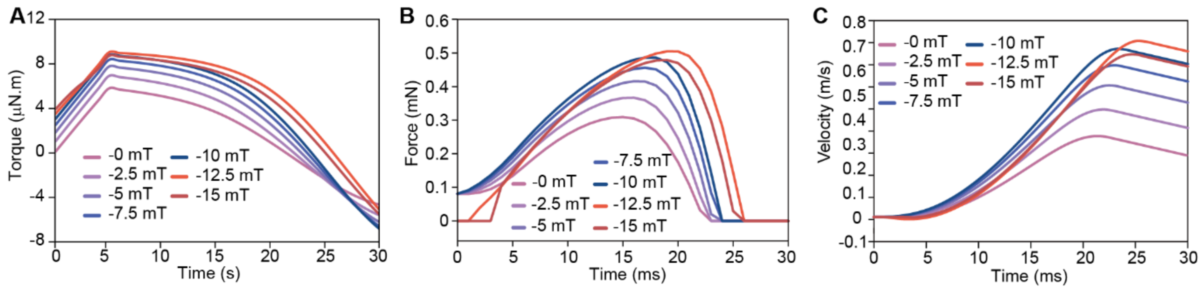


**Figure S16.**

**The jumping performance of the robot with different initial magnetic fields.** A-C) The torque profiles (A), force profiles (B), and velocity profiles (C) obtained from the numerical simulation corresponding to the jumping processes with different initial magnetic fields.

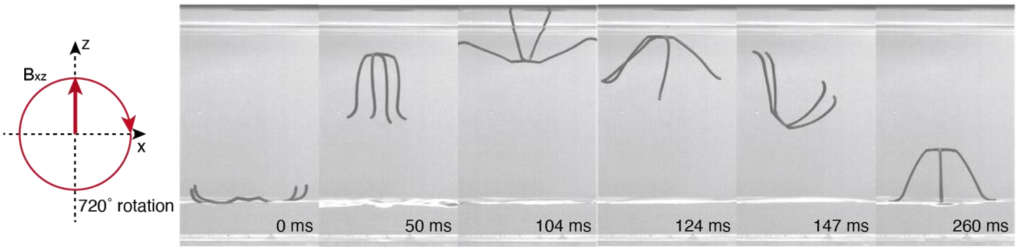


**Figure S17.**

**The robot performs a 720° backflip in the flight phase.** The robot performs in-flight 720° backflip actuated by a rotating magnetic field (scale bar, 1 cm). The amplitude of the rotating magnetic field is 20 mT, and the rotating frequency is 20 Hz.

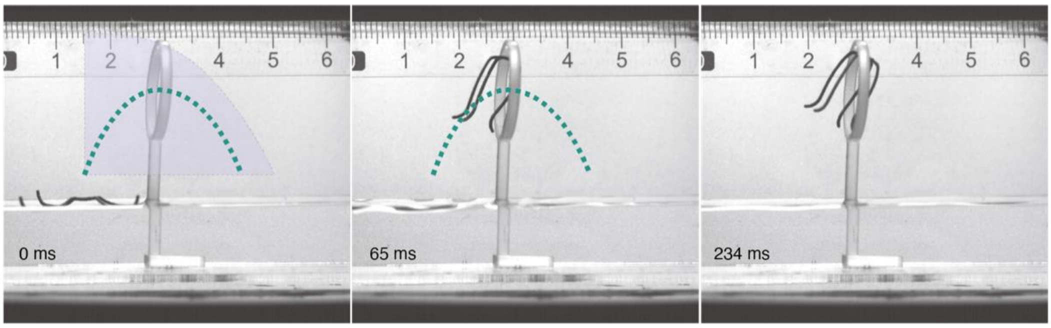


**Figure S18.**

**The robot fails to pass through a ring-like obstacle without pose control.** The purple region is the maximum accessible region of the robot via a forward jumping. The green dashed line is the planned trajectory.

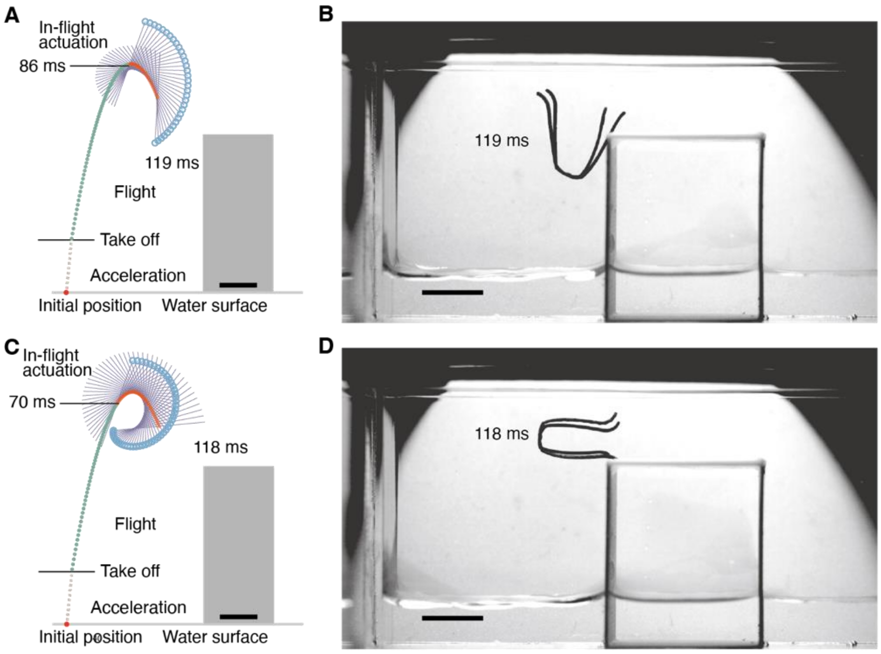


**Figure S19.**

**The controllable energy restoration of the robot by adjusting the starting time of the in-flight actuation.** A) The predicted trajectory and pose of the robot when the in-flight actuation starts at 86 ms (scale bar, 5 mm). B) The robot comes into contact with the obstacle with a pose angle of 180°, when the in-flight actuation starts at 86 ms (scale bar, 1 cm). C) The predicted trajectory and pose of the robot when the in-flight actuation starts at 70 ms (scale bar, 5 mm). D) The robot comes into contact with the obstacle with a pose angle of 270°, when the in-flight actuation starts at 70 ms (scale bar, 1 cm).

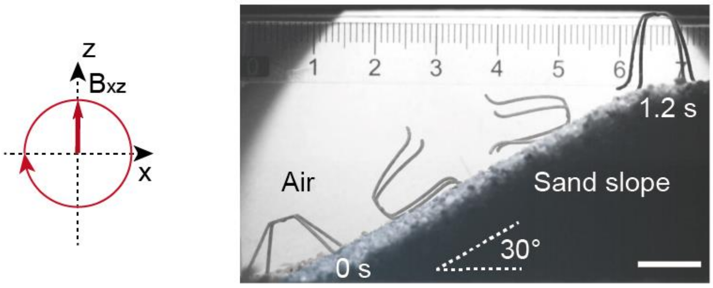


**Figure S20.**

**The robot tumbles on a sand slope.** The robot tumbles along a sand slope actuated by a rotating magnetic field with a strength of 10 mT and a frequency of 2 Hz. The tilted angle of the slope is 30°.

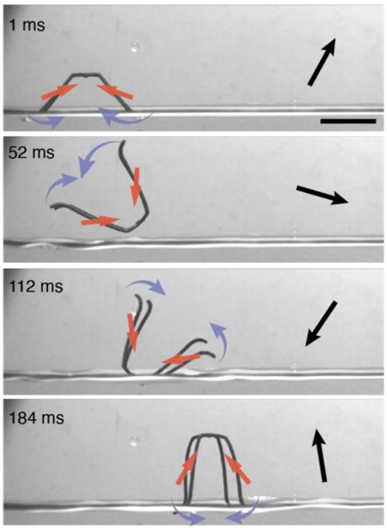


**Figure S21.**

**Gait analysis of the tumbling motion on the water surface.** The tumbling locomotion is actuated by a rotating magnetic field with a strength of 10 mT and a frequency of 5 Hz. The black arrows indicate the directions of the magnetic fields, the red arrows indicate the magnetization directions, and the purple arrows indicate the torques exerted on the robot legs.

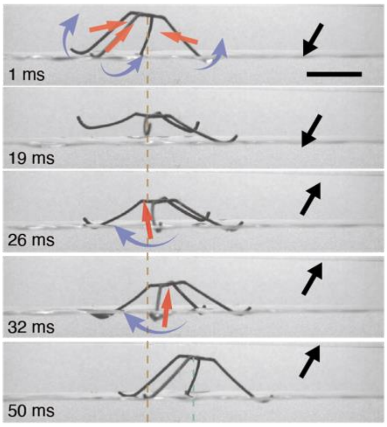


**Figure S22.**

**Gait analysis of the paddling locomotion.** The paddling locomotion is actuated by a one-dimensional oscillating magnetic field with an amplitude of 4 mT and a frequency of 20 Hz. The black arrows indicate the directions of the magnetic fields, the red arrows indicate the magnetization directions, and the purple arrows indicate the torques exerted on the robot legs.

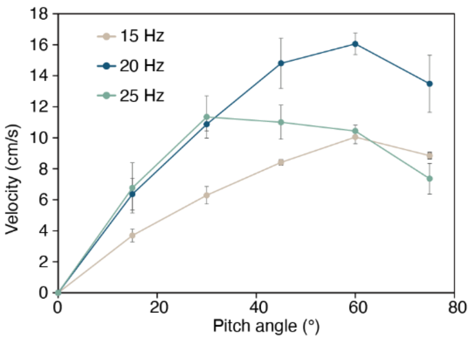


**Figure S23.**
**The velocity of the paddling locomotion.**

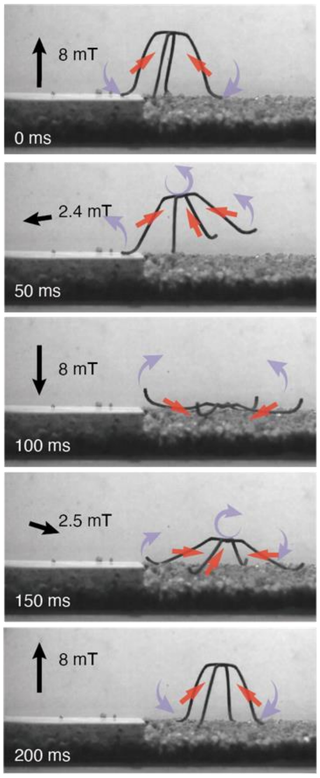


**Figure S24.**

**Gait analysis of the walking locomotion.** The walking locomotion is actuated by an elliptically rotating magnetic field. The black arrows indicate the directions of the magnetic fields, the red arrows indicate the magnetization directions, and the purple arrows indicate the torques exerted on the robot legs.

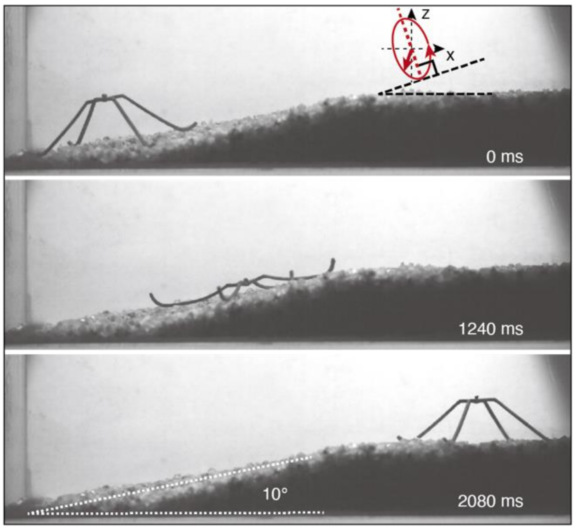


**Figure S25.**

**The robot walks on a sand slope.** The walking locomotion is actuated by an elliptically rotating magnetic field. The long axis of the elliptically rotating magnetic field is perpendicular to the slope.

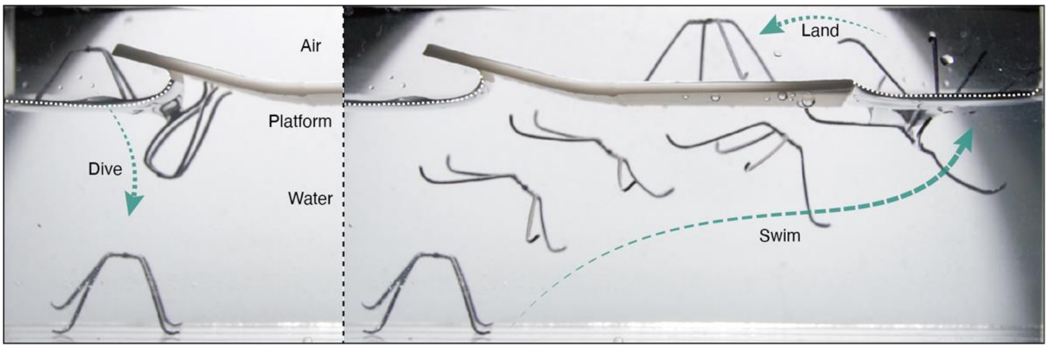


**Figure S26.**

**Locomotion of the robot in an aquatic-terrestrial environment.** The robot is actuated to make adaptive locomotion across from the water surface to underwater, and then travels back to the stage.

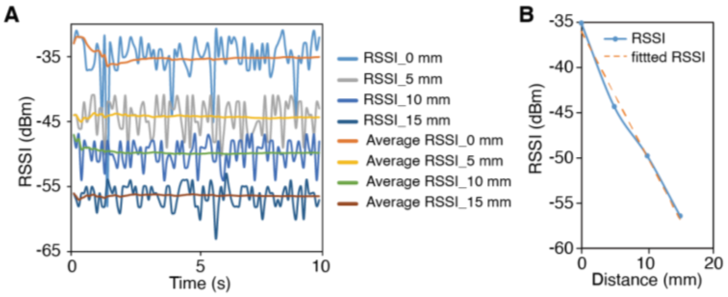


**Figure S27.**

**The received signal strength indicator (RSSI) as a function of distance between the antenna and the RFID tag.** A) The RSSI detected by the antenna when the RFID tag is placed at different distances from the antenna. B) The RSSI as a function of distance between the antenna and the RFID tag.

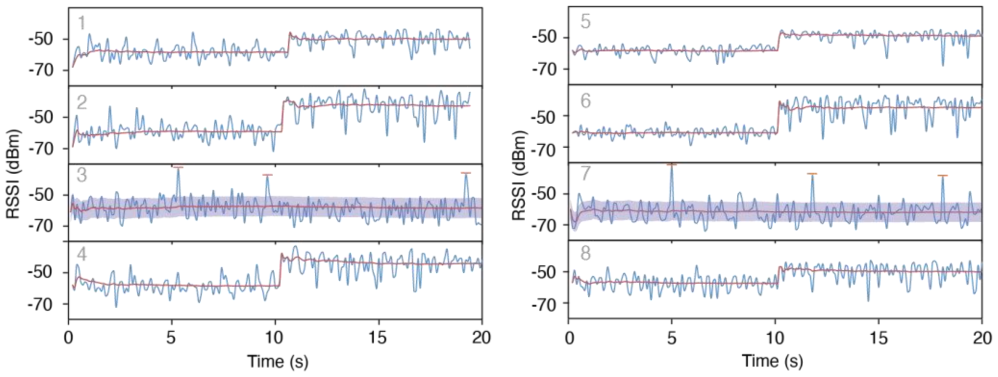


**Figure S28.**
The received signal strength indicator (RSSI) measured at different positions of the maze.

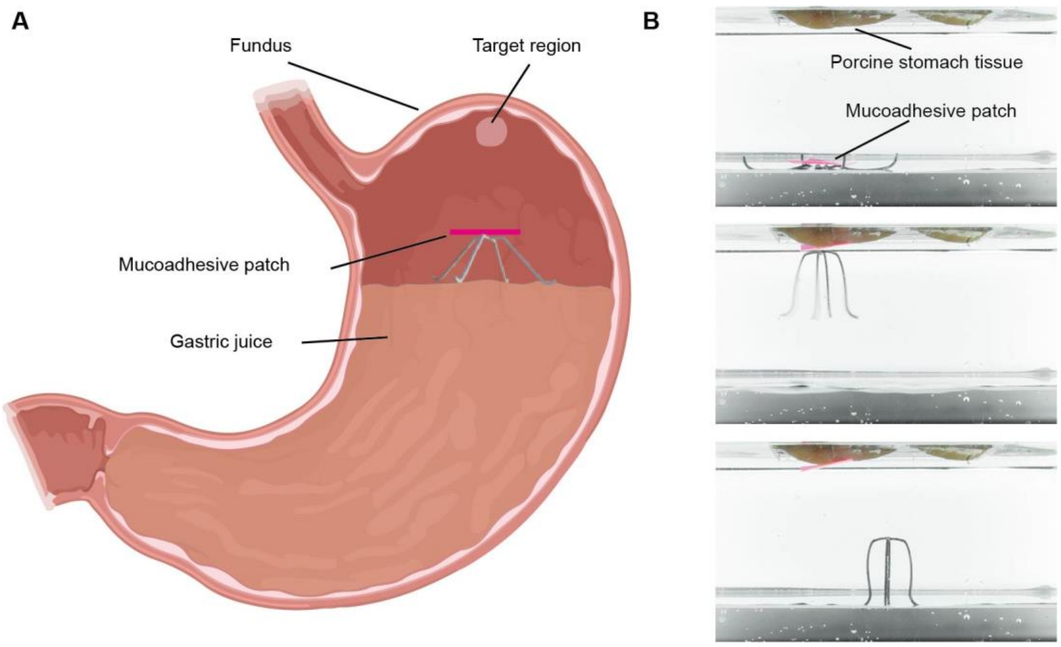


**Figure S29.**

**Towards potential biomedical application.** A) Schematics of a possible scenario where the jumping robot can be implemented. B) The robot delivers a mucoadhesive patch to a piece of porcine stomach tissue.

**Table S1.**

The summary of the jumping performances and the designed parameters of jumping robots on the water surface.

| Body length L (mm) | Mass (mg) | Jumping height H (mm) | H/L | Ref. |
|---|---|---|---|---|
| 250 | 11000 | 140 | 0.56 | (*17*) |
| 100 | 12500 | 95 | 0.95 | (*19*) |
| 74 | 510 | 26 | 0.35 | (*16*) |
| 102 | 68 | 142 | 1.39 | (*8*) |
| 25 | 33 | 30 | 1.2 | this work |
| 25 | 33 | 38 | 1.52 | this work |

**Movie S1.**

This movie sequentially shows the robot jumping on the water surface (Fig. 1E), jumping on the solid ground (Fig. 1F), and jumping forward with a desired direction on the water surface (Fig. 1I).

**Movie S2.**

This movie sequentially shows the in-flight 360° backflip (Fig. 3A) and in-flight 720° backflip (Fig. S17) of the robot on the water surface, and the robot jumping through a ring-like obstacle (Fig. 3C), jumping over a wall-like obstacle (Fig. 3D), jumping over a high stage (Fig. 3F), and landing on a high stage (Fig. 3G) with the programmed in-flight maneuver.

**Movie S3.**

This movie sequentially shows the robot swimming in the water (Fig. 4A,B), tumbling on a sand slope (fig. S20), tumbling on the water surface (Fig. S21), paddling on the water surface (Fig. 4D), and walking on the ground and sand (Fig. 4E).

**Movie S4.**

This movie sequentially shows the robot integrated with a soft gripper picking, transporting, and releasing a non-magnetic cargo (Fig. 5A), and the robot integrated with a needle performing targeted puncturing (Fig. 5B).
